# The association between economic indicators and the incidence of tetraplegia from traumatic spinal cord injury in Taiwan

**DOI:** 10.1186/s12883-021-02141-8

**Published:** 2021-03-17

**Authors:** Wei-Chih Lien, Wei-Ming Wang, Jung-Der Wang, Fuhmei Wang

**Affiliations:** 1 Department of Physical Medicine and Rehabilitation, National Cheng Kung University Hospital, College of Medicine, National Cheng Kung University, Tainan, 704 Taiwan; 2 Department of Physical Medicine and Rehabilitation, College of Medicine, National Cheng Kung University, Tainan, 701 Taiwan; 3 Ph.D. Program in Tissue Engineering and Regenerative Medicine, National Chung Hsing University, Taichung, 402 Taiwan; 4 Department of Statistics, College of Management, National Cheng Kung University, Tainan, 701 Taiwan; 5 Department of Public Health, College of Medicine, National Cheng Kung University, Tainan, 701 Taiwan; 6 Departments of Internal Medicine and Occupational and Environmental Medicine, National Cheng Kung University Hospital, Tainan, 704 Taiwan; 7 Department of Economics and Department of Public Health, National Cheng Kung University, Tainan, 701 Taiwan

**Keywords:** Incidence, Spinal cord injuries, Tetraplegia, Gross domestic product, Income elasticity, Motor vehicle injury

## Abstract

**Background:**

Economic performance may affect public health parameters. This study aimed to determine the time trend of incidence of traumatic spinal cord injury (SCI) and its association with income, presented by GDP (gross domestic product) per capita.

**Methods:**

This study was a retrospective observational study in Taiwan. Newly diagnosed SCI patients with moderate to severe disability from 2002 to 2015 were identified from the reimbursement database of the National Health Insurance (NHI) system (1998–2015). CIR_16–99_ (cumulative incidence rate, aged 16–99 years, per 10^3^ person-years) and CIR_16–59_ (aged 16–59 years) of SCI from 2002 to 2015 were measured.

**Results:**

There were 5048 newly diagnosed SCI patients during the study period. After controlling the factors of sex, urbanization level, literacy, income inequality, and global financial crisis (mixed effects models), the CIR_16–99_ of SCI, traumatic SCI, motor vehicle (MV)-related SCI, fall-related SCI, tetraplegia, traumatic tetraplegia, MV-related tetraplegia, and fall-related tetraplegia were inversely associated with GDP per capita; the *β* coefficients ranged from − 4.85 (95% confidence interval − 7.09 to − 2.6) for total SCI to − 0.8 (− 1.3 to − 0.29) for fall-related tetraplegia. We restricted our comparison to Taipei City and the 4 lowest densely populated counties, which also corroborated with the above results. The income elasticity analysis revealed when GDP per capita increased by 1%, the total SCI decreased by 1.39‰; which was also associated with a decrease of 1.34‰, 1.55‰, 1.36‰, 1.46‰, 1.54‰, 1.54‰, and 1.62‰ for traumatic SCI, MV-related SCI, fall-related SCI, tetraplegia, traumatic tetraplegia, MV-related tetraplegia, and fall-related tetraplegia respectively. The *β* coefficients show that the compared areas of urbanization level were also inversely correlated with CIR_16–59_ in the SCI population.

**Conclusions:**

We conclude that the incidence of tetraplegia of traumatic SCI in Taiwan decreases with good economic performance, which may be resulted from the provision of public goods and services, possibly through improvements in the infrastructure of transportation and construction.

**Supplementary Information:**

The online version contains supplementary material available at 10.1186/s12883-021-02141-8.

## Background

Traumatic spinal cord injury (SCI) has profound physical, social, and economic impacts for the individual and society [1]. Although neurorestorative strategies, for example cytotherapy, have been shown to be beneficial, there is no effective treatment to restore neurological impairments after SCI [2]. The findings of epidemiological studies of SCI over time across regions have revealed dissimilar results. In Galicia, Spain, the overall incidence rate (IR) of traumatic SCI was 23.4 per 1 million people in 1995 and dropped down to 1.4 in 2014 [3]. In the United States (US), the incidence rate of traumatic SCI was 53 per 1 million people in 1993 and slightly went up to 54 per 1 million people in 2012 [4]. In Taiwan, the annual IR of SCI was 24.5 per 1 million people in 1993 and dropped down to 17.2 in 1996 [5].

Motor vehicle (MV) traffic accidents were the primary etiology of traumatic SCI among developed countries (43–57.3%), including the US and some OECD (Organization for Economic Co-operation and Development) countries, such as Japan, the United Kingdom (UK), Portugal, Australia, and Italy [6–11]. In Fiji, Romania, and Russia, falls were the primary cause of SCI [12–14]. In other countries, falls were usually the second major cause of SCI [15]. MV accidents and falls frequently resulted in tetraplegia [16]. In California, the incidence rate of MV-related SCI gradually decreased during 1996–2008, which may be associated with the improvements in public health campaigns and road traffic safety [17]. In Taiwan, motor vehicle traffic accidents were also the primary etiology of SCI cases, and about half of them (49.9%) had cervical injury [5]. In Australia, the proportion of tetraplegia among SCI was 57.7% [9]. Tetraplegia resulted in higher loss of life expectancy, especially in young and middle-aged adults [18], and affected quality of life to a greater extent [19].

**Fig. 1:**
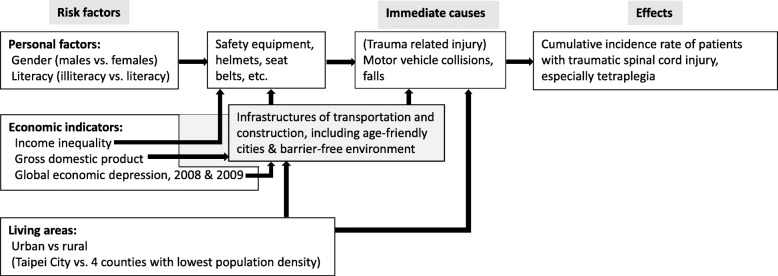
Possible causal diagram illustrates the hypothetical association between risk factors and traumatic spinal cord injury, including tetraplegia

Economic development usually leads to improvements in health parameters which has been shown in population-based studies [20, 21]. According to the World Bank Country and Lending Groups, the overall incidence rate of traumatic spinal injury in high income countries, such as the US, Sweden, Canada, Finland, Australia, Spain, Japan, and Taiwan, seemed to be lower in comparison with low and middle income countries, such as Turkey, Nigeria, Nepal, Malaysia, India, Brazil, Iran, China, South Africa, and Mexico [22]. Gross domestic product (GDP) exhibits the market value of the aggregate goods and services produced within a country in a given time period and is similar to a barometer of a nation’s economy. GDP per capita measures an individual’s living standard and could provide us with past trends, current patterns, and future predictions. Previous studies revealed that illiteracy [23] and income inequality [24], old age [25], and residing in rural area [26] were related to fatal traumatic injuries, indicating the need for control of these confounding factors, as illustrated in Fig. 1. However, to the best of our knowledge, no study has explored the association between the IRs of SCI and living standard, presented by GDP per capita, longitudinally. Therefore, the objectives of this study were to assess the trends of etiology in IRs, the association between IRs and GDP per capita, and the influence of rural-urban disparity in tetraplegia of acute traumatic SCI from 2002 to 2015 in Taiwan.

## Methods

This study was first approved by the Institutional Review Board of National Cheng Kung University Hospital, College of Medicine, National Cheng Kung University before commencement (Approval No.: B-ER-105-386). Because this study analyzed national reimbursement data plus related secondary data, of which all personal identification information was fully encrypted, it did not require patient’s informed consent according to our national regulation of Personal Data Protection Act (https://law.moj.gov.tw/ENG/LawClass/LawAll.aspx?pcode=I0050021).

### Study design and identification of patients with traumatic SCI

**Fig. 2:**
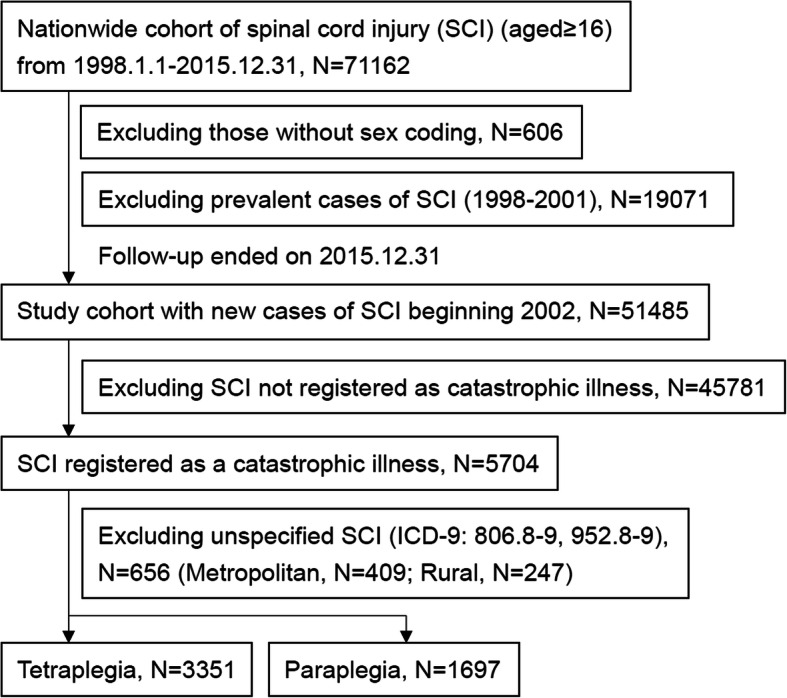
Flowchart showing the selection of spinal cord injury (SCI) cohort

In Taiwan, the coverage of health insurance and access to healthcare are prevalent and boundless for every citizen, and provided by the governmental agency of NHI (National Health Insurance) Administration. Because diagnostic accuracy is usually a consideration with claim data sets, the NHI Administration adopts regular evaluation of medical records to make certain of the information. Deceitful recording will result in heavy penalties and audits are conducted for both hospitals and physicians involved. In this study, we used ICD-9-CM diagnosis codes of SCI with fracture of vertebral column (coding 806) and SCI without evidence of spinal bone injury (coding 952) to represent the SCI population in nationwide health insurance claims data from 1998 to 2015 [27]. These two ICD-9-CM codes (806 and 952) were applied for identifying traumatic SCI based on the“Uniform Data Systems Cases Definition” suggested by the US Centers for Disease Control and Prevention [28]. All patients ≥16 years of age with newly diagnosed SCI were included. The index date was the date of the first hospitalization due to SCI within the period from January 1, 2002 to December 31, 2015. The levels of injury were divided into cervical (806.0–1, 952.0), thoracic (806.2–3, 952.1), lumbar (806.4–5, 952.2), and sacral (including cauda equine syndrome), excluding unspecified levels (806.8–9, and 952.8–9). Due to the sparse number of cases, cervical SCI was designated as tetraplegia, and the latter three (i.e. thoracic, lumbar, and sacral levels) were combined for analysis and categorized as paraplegia and/or cauda equine syndrome [27]. The archives of catastrophic illness patients collated by the NHI Administration were used to represent patients with SCI resulting in moderate and severe disability (*N* = 5704). Because all patients successfully registered in this registry are waived from all copayment, the NHI Administration requires two specialists to validate the persistent functional disability after SCI [29]. Six hundred and fifty six patients with injury at unspecified levels were excluded as mentioned above. Among the 5048 patients with SCI, 3351 patients were recorded as tetraplegia (Fig. 2) and external codes were further abstracted to identify the mechanisms of injury. The E-codes (external cause of injury) included in our review were E810–E819 (MV traffic accidents), fall (E880–E888), and others (the rest of the E-codes). Urbanization levels of acute admissions were divided into metropolitan areas with satellite cities and rural areas [30]. Thus, we stratified all patients with SCI and tetraplegia by gender, age, E-code, and urbanization level.

Since patients successfully registered on the list of catastrophic illnesses can be waived from co-payment, every patient is required to be validated by two physicians to prevent abuse. The only chance of miscalculation of the incidence rate of disabled patients with SCI was if an event happened before 1996 or prior to the establishment of the reimbursement database on January 1, 1998. To avoid including prevalent cases, a previous study documents that at least a 36-month prevalence period should be allowed to exclude previously diagnosed patients [31]. We thus excluded data from the first 4 years (1998–2001) to assure that all included cases were incident cases.

We also abstracted the following major comorbidities: including stroke (ICD-9-CM: 430–437), acute myocardial infarction (AMI, ICD-9-CM: 410), chronic obstructive pulmonary disease (COPD, ICD-9-CM: 491–492), liver cirrhosis (ICD-9-CM: 571.2, 571.5, 571.6), end-stage renal disease (ESRD, ICD-9-CM: 585), and cancer (ICD-9-CM: 140–208). Then, the Charlson Comorbidity Index (CCI) [32] was calculated to explore if there were any specific trends in patients with SCI.

### Estimating the cumulative incidence rates (CIRs) of SCI

In general, the peak ages for SCI were 16–30 years and 45–59 years, during their working age [15, 33], and the majority of fall-related traumatic SCI occurred in elderly people. We therefore used two age ranges 16–99 and 16–59 for every consecutive calendar year between 2002 and 2015 to determine if both were associated with the economic indicators. The CIR per 10^3^ person-years was calculated from 16 to 99 year-old patients to estimate the CIR_16–99_ of all SCI, and also from 16 to 59 year-old patients to estimate the CIR_16–59_ of young and middle-aged patients with SCI [11, 34].

### Mixed effects model for nationwide data of SCI

GDP per capita (US dollars in 2011) [35] in natural logarithm form (Ln (GDP per capita)) was the independent variable for presenting the changes of income or the economic growth rate. Taking into account the global financial crisis between 2008 and 2009, a dummy year was used, whereby the calendar years 2008 and 2009 were individually designated as 1, and other years as 0. Moreover, we applied the literacy rate at 20–64 years (LR_20–64_) [36] and the ratio of the average income of the richest 20% to the poorest 20% (P80/P20) [37], to account for potential confounding of literacy and income inequality. We used mixed effects models to analyze the association between the CIRs of SCI and Ln (GDP per capita) for nationwide patients with SCI (*Y*_*n*_; *n* = 1–8; *Y*_*1*_ = total SCI, *Y*_*2*_ = traumatic SCI, *Y*_*3*_ = MV-related SCI, *Y*_*4*_ = fall-related SCI, *Y*_*5*_ = tetraplegia, *Y*_*6*_ = traumatic tetraplegia, *Y*_*7*_ = MV-related tetraplegia, *Y*_*8*_ = fall-related tetraplegia), where *β*_*0*_ is constant, *β*_*s*_ (s = 1–7) are coefficients to adjacent variables, and ε is the approximation error.

The regression model is as follows:

1 $$ {Y}_n={\beta}_0+{\beta}_1 sex+{\beta}_2 urbanization+{\beta}_3 year2008+{\beta}_4 year2009+{\beta}_5 Ln\left( GDP\  per\  capita\right)+{\beta}_6{LR}_{20-64}+{\beta}_7P80/P20+\varepsilon $$

### Mixed effects model of SCI in Taipei City and the 4 counties with the lowest population density (Taitung County, Yilan County, Hualien County, and Nantou County)

There were 4 counties with a population density lower than 220/km^2^ from 2002 to 2015 in Taiwan, namely Taitung County, Yilan County, Hualien County, and Nantou County. We constructed mixed effects models using the same equation as Eq. (1) to analyze the association between the CIRs of SCI and Ln (GDP per capita) for patients with SCI in Taipei City (the capital and a special municipality of Taiwan with a population density above 9500/km^2^) in contrast to the 4 counties with the lowest population density as a cluster to corroborate the robustness of our models.

### The measurement of income elasticity

The income elasticity of SCI was estimated according to Eq. 2, as shown by Newhouse in the 1970s [38].

2 $$ \frac{\partial {CIR}_{16-99}}{\partial \ln \left( GDP\  per\  capita\right)}\times \frac{1}{CIR_{16-99}} $$

### Statistical analyses

We used mixed-effects models to examine the relationship between CIR and Ln (GDP per capita) in SCI and tetraplegia. Differences between the characteristics of tetraplegia and traumatic tetraplegia were evaluated utilizing an independent *t* test for continuous variables and the *χ*^*2*^ test for nominal variables. *β* coefficient, 95% confidence interval (CI), and *p* value were calculated in the mixed effects model. A *p* value of < 0.05 was regarded as indicating statistical significance. All statistical analyses were completed using the statistical package SAS (Version 9.3, SAS Institute, Cary, NC).

## Results

In total, 5048 newly diagnosed patients with SCI from 2002 to 2015 were included in this study, with 3351 tetraplegia and 2182 traumatic tetraplegia patients. The mean ages of all SCI patients, tetraplegia, and traumatic tetraplegia were 50.3 (95% CI 49.8 to 50.8), 52.4 (51.9 to 52.9), and 52.2 (51.5 to 52.9) years, respectively. About four-fifths were males. Among all patients with SCI, 19.0% were in the dependent insurance group; 30.1% had yearly income less than the median level (640 US dollars), and 50.9% had yearly income greater than the median level. 49.4% of the SCI incidents and 51.4% of the tetraplegia incidents occurred in rural areas. Among all patients with SCI, about 56.7% scored 0 on the CCI [32], 17.9% scored 1, and the remaining 25.4% scored > 1. The proportion of patients with chronic obstructive pulmonary disease was higher in tetraplegia than in traumatic tetraplegia. The proportion of multiple comorbidities (CCI > 1) was higher in tetraplegia than in traumatic tetraplegia (Table 1).

**Table 1:** Background characteristics for total spinal cord injury (SCI), tetraplegia and traumatic tetraplegia cases in Taiwan, 2002–2015

Characteristic	SCI, N (%)	Tetraplegia, N (%)	Traumatic tetraplegia, N (%)
Total number of subjects	5048 (100%)	3351 (100%)	2182 (100%)
Age, mean (range), years^a^	50.3 (range 16–96)	52.4 (range 16–96)	52.2 (range 16–94)
Age group^b^			
16–39	1328 (26.3%)	704 (21.0%)	457 (20.9%)
40–59	2198 (43.5%)	1501 (44.8%)	995 (45.6%)
60–99	1522 (30.2%)	1146 (34.2%)	730 (33.5%)
Sex^b^			
Male	3927 (77.8%)	2738 (81.7%)	1786 (81.9%)
Female	1121 (22.2%)	613 (18.3%)	396 (18.1%)
Degree of urbanization^b^			
Metropolitan areas including satellite cities	2556 (50.6%)	1629 (48.6%)	1017 (46.6%)
Rural areas	2492 (49.4%)	1722 (51.4%)	1165 (53.4%)
Monthly insured salary (US dollar)^b^			
Dependent	961 (19.0%)	840 (25.1%)	380 (17.4%)
< 640 US dollars	1517 (30.1%)	824 (24.6%)	627 (28.7%)
≥ 640 US dollars	2570 (50.9%)	1687 (50.3%)	1175 (53.9%)
Major comorbidities			
Stroke (ICD-9-CM: 430–437)^b^	315 (6.2%)	218 (6.5%)	115 (5.3%)
Acute myocardial infarction (ICD-9-CM: 410)^b^	25 (0.5%)	17 (0.5%)	6 (0.3%)
COPD (ICD-9-CM: 491–492)*^b^	179 (3.6%)	119 (3.6%)	55 (2.5%)
Liver cirrhosis (ICD-9-CM: 571.2, 571.5, 571.6)^b^	80 (1.6%)	58 (1.7%)	39 (1.8%)
ESRD (ICD-9-CM: 585)^b^	86 (1.7%)	49 (1.5%)	25 (1.2%)
Cancer (ICD-9-CM: 140–208)^b^	116 (2.3%)	80 (2.4%)	47 (2.2%)
Charlson Comorbidity Index*^b^			
0	2863 (56.7%)	1989 (59.4%)	1355 (62.1%)
1	905 (17.9%)	655 (19.6%)	429 (19.7%)
> 1	1280 (25.4%)	707 (21.1%)	398 (18.2%)

*CI* confidence interval, *ICD-9-CM* International Classification of Disease, Ninth Revision, Clinical Modification, *COPD* chronic obstructive pulmonary disease, *ESRD* end-stage renal disease

*p < 0.05, comparison between tetraplegia and traumatic tetraplegia

^a^The independent *t* test

^b^The *χ*^*2*^ test

**Fig. 3:**
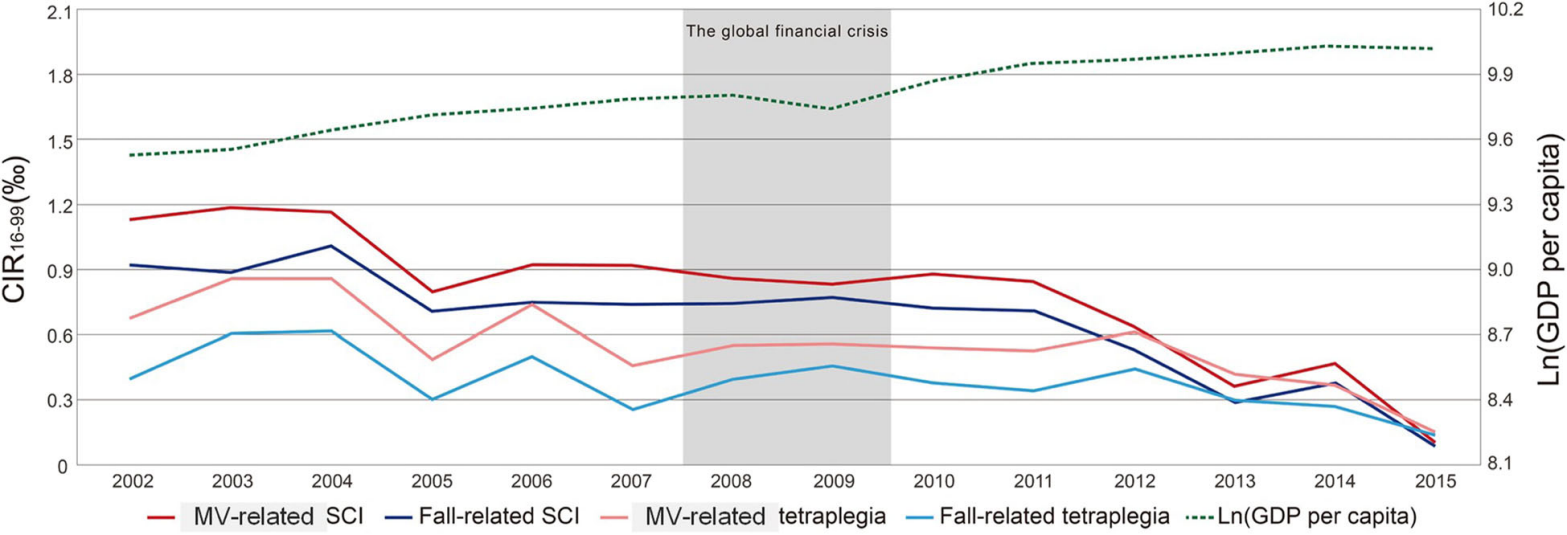
Time trends of CIR_16–99_ (cumulative incidence rate per 10^3^ person-years, aged 16–99) and GDP (gross domestic products) per capita (US dollars in 2011) in natural logarithm (Ln) form from 2002 to 2015 for motor vehicle (MV)-related SCI (spinal cord injury), fall-related SCI, MV-related tetraplegia, fall-related tetraplegia

Figure 3 shows the trends for the various types of SCI incidence and GDP per capita in natural logarithm form. The majority of external causes of injury were MV collisions followed by falls during 2002–2011. Beginning 2012, however, the CIR_16–99_ of MV-related SCI decreased rapidly and appeared close to those of fall-related SCI and tetraplegia in 2015.

After controlling for sex, urbanization level, global financial crisis (year 2008, 2009), literacy rate, and income inequality (mixed effects model), we found the CIR_16–99_ of SCI, traumatic SCI, MV-related SCI, fall-related SCI, tetraplegia, traumatic tetraplegia, MV-related tetraplegia, and fall-related tetraplegia were all inversely correlated with Ln (GDP per capita); the *β* coefficients ranged from − 4.85 (95% CI − 7.09 to − 2.6) for total SCI to − 0.8 (− 1.3 to − 0.29) for fall-related tetraplegia (Table 2). The mixed effects models constructed for Taipei City and the 4 least densely populated counties in Taiwan showed similar results of consistent inverse correlations between CIR and Ln (GDP per capita), with *β* coefficients ranging from − 7.23 (95% CI − 11.2 to − 3.26) for total SCI to − 1.91 (− 3.37 to − 0.45) for MV-related tetraplegia, as summarized in Table 2. We additionally tested the robustness of the above association between the CIR of those aged 16–59 and Ln (GDP per capita); the *β* coefficients of the areas of acute admission were also inversely correlated with CIR_16–59_ in all SCI populations (Supplementary Table 1).

**Table 2:** The CIR_16–99_ (cumulative incidence rate per 10^3^ person-years, aged 16–99) of spinal cord injury (SCI) and major risk factors in Taiwan from 2002 to 2015

Characteristic	SCI	Tetraplegia	Traumatic tetraplegia
CIR_16–99_, mean (95% CI), ‰*^a^	3.5 (2.9 to 4.2)	2.5 (2.0 to 2.9)	1.6 (1.3 to 1.9)
Literacy rate, %	99.4 (99.2 to 99.6)		
P80/P20^b^	7.57 (7.45 to 7.7)		
GDP^b^, mean (95% CI), US dollars	18,447 (16,862 to 20,032)		
Ln (GDP^b^ per capita), mean (95% CI), US dollars	9.8 (9.7 to 9.9)		

*CI* confidence interval

*p < 0.05, comparison between tetraplegia and traumatic tetraplegia

^a^The independent *t* test

^b^P80/P20: the ratio of the average income of the richest 20% to the poorest 20%; GDP: gross domestic product

The incidence of SCI in Taiwan decreased from 5‰ in 2002 to 1‰ in 2015. GDP per capita grew steadily about 63% over this period, as Fig. 3 presents. This brief deviation in various SCI suggests that incidence rates turn out to be strongly connected to GDP per capita. The extent of the changes in SCI decreases with GDP per capita. Based on the mean of CIR_16–99_ of total SCI in Table 2 and Eq. (2), the income elasticity of total SCI related to GDP per capita is − 1.39. Namely, when GDP per capita grows by 1%, total SCI decreases by 1.39‰. Similarly, as GDP per capita increases by 1%, the incidence rates of traumatic SCI, MV-related SCI, fall-related SCI, tetraplegia, traumatic tetraplegia, MV-related tetraplegia, and fall-related tetraplegia decrease by 1.34‰, 1.55‰, 1.36‰, 1.46‰, 1.54‰, 1.54‰, and 1.62‰, respectively (Supplementary Table 2).

## Discussion

The objectives of this study were to explore the associations between economic indicators and occurrence of SCI. Although we found a consistent inverse correlation between the CIR_16–99_ of various types of SCI and Ln (GDP per capita), it does not necessarily indicate that it is causally related. We have presented, however, the following arguments to corroborate the above hypothesis: First, this is a longitudinal nation-wide cohort study, which involved an unbiased participant selection process and accurately assessed the incidence rates of SCI based on all new cases in the catastrophic registry. Since the registry of every patient requires a board-certified physician to provide sufficient evidence plus another physician assigned by the NHI Administration to validate it, we are confident that the diagnosis is accurate to avoid any abuse of waiving copayment. Second, since we have comprehensively controlled the major risk factors for the occurrence of SCI (Fig. 1), including age, sex, urban-rural disparity, literacy rate, income inequality, and years with major economic depression (the global financial crisis) in the construction of mixed effects models, plus we stratified the patients into different injury levels and external causes, these factors cannot be explanatory to the independent association between improved economic performance and decreased occurrence of SCI. Third, we have tested the robustness of these models by restrictive comparison between Taipei City and the 4 counties with the lowest population density, which still corroborates our hypothesis. Therefore, we tentatively conclude that the inverse association between GDP per capita and incidence of SCI (Fig. 3) might be causal and deserves attention.

The *β* coefficients of Ln (GDP) were higher in the 16–99 year age group of all SCI than in the 16–59 year age group. Increases in GDP per capita also seemed to have preventive effects on old people for the occurrence of SCI. In addition, residing in metropolitan areas also showed a preventive effect in lowering the occurrence of all SCI, but such an effect appeared to be diminished in the occurrence of fall-related tetraplegia among young and middle aged people. This may be due to the different mechanisms of fall-related tetraplegia in old versus young to middle aged populations, as the latter often occurs due to occupational or sport-related injuries [39].

Our results corroborate previous studies that show MV accidents are the most prevalent etiology of SCI [40]. The inverse association between incidence rate of MV-related SCI and GDP per capita in Taiwan could be resulted from improvements in both transportation infrastructure and accessibility of public transportation in urban regions. Economies with higher GDP per capita accumulate more tax revenue to be able to establish better public transportation systems than those with lower GDP per capita. Among them, South Korea is one of these examples, which shows a high association between increase in GDP and increase in capital of the transportation sector [41]. Moreover, our study found that urbanization was an independent factor for reduction in the occurrence of SCI (Table 3). In fact, the increased usage of public transportation from 16.3% in 2009 to 18.0% in 2015 may have contributed to the lower occurrence of SCI in Taiwan [42]. The public transportation system in urban regions of Taiwan is predominated by bus and railway systems, and Taipei City’s transportation system is superior to those of all other areas in Taiwan. Taipei City established the first medium-capacity metro line in March 1996 and the system quickly expanded to cover the whole metropolitan area [43]. Public transport usage rate in Taipei City was 40% in contrast to below 10% for the 4 counties with the lowest population density (Yilan County, Taitung County, Hualien County, and Nantou County) during 2009–2015 [42]. Moreover, the number of heavy-type motorcycles per 100 people in Taipei City was much lower compared to those of the 4 counties with the lowest population density during 2002–2015 [44]. Motorcycle accidents are also a growing hazard of a cervical cord injury [26], which may be why the occurrences of traumatic SCI, tetraplegia, and traumatic tetraplegia in urbanized Taipei City versus the 4 counties are about 1.5–2 times lower than those of the whole country (Table 3). Simultaneously, the potential effect of income inequality in Taipei City has become less significant, probably due to the high accessibility of convenient public transportation and excellent enforcement on safety regulations, such as penalties for drink driving and other traffic offences.

**Table 3:** Estimation results of constructed mixed-effects model with CIR_16–99_^a^ of spinal cord injury (SCI) as the dependent variable and major risk factors as fixed effects

	SCI	Traumatic SCI	MV^a^-related SCI	Fall-related SCI	Tetraplegia	Traumatic tetraplegia	MV^a^-related tetraplegia	Fall-related tetraplegia
National data								
Sex (Male/Female)	1.85^***^ (1.59 to 2.1)	1.21^***^ (1.01 to 1.4)	0.62^***^ (0.5 to 0.73)	0.37^***^ (0.29 to 0.44)	1.56^***^ (1.35 to 1.77)	1.01^***^ (0.85 to 1.18)	0.55^***^ (0.44 to 0.65)	0.29^***^ (0.23 to 0.34)
Ln (GDP^a^ per capita), USD	−4.85^***^ (−7.09 to −2.6)	−3.07^***^ (− 4.78 to −1.36)	− 1.55^**^ (− 2.56 to −0.55)	− 1.09^***^ (− 1.69 to − 0.48)	− 3.64^***^ (−5.51 to − 1.78)	−2.46^**^ (− 3.9 to − 1.02)	− 1.23^*^ (− 2.15 to − 0.31)	− 0.8^**^ (− 1.3 to − 0.29)
Urbanization level (Metropolitan/Rural)	−0.65^***^ (− 0.9 to − 0.39)	−0.56^***^ (− 0.76 to − 0.37)	−0.31^***^ (− 0.42 to − 0.2)	−0.14^***^ (− 0.21 to − 0.07)	−0.49^***^ (− 0.7 to − 0.28)	−0.46^***^ (− 0.62 to − 0.29)	−0.29^***^ (− 0.39 to − 0.18)	−0.1^***^ (− 0.16 to − 0.04)
Year 2008 (Yes/No)	−0.34 (− 0.91 to 0.23)	−0.32 (− 0.75 to 0.11)	−0.1 (− 0.35 to 0.16)	−0.13 (− 0.29 to 0.02)	−0.24 (− 0.72 to 0.23)	−0.22 (− 0.58 to 0.15)	−0.08 (− 0.31 to 0.16)	−0.1 (− 0.23 to 0.03)
Year 2009 (Yes/No)	−1.49^**^ (− 2.47 to − 0.52)	−1.13^**^ (− 1.87 to − 0.38)	−0.54^*^ (− 0.98 to − 0.1)	−0.3^*^ (− 0.57 to − 0.03)	− 1.13^**^ (− 1.94 to − 0.31)	−0.77^*^ (− 1.39 to − 0.14)	−0.43^*^ (− 0.83 to − 0.03)	−0.16 (− 0.38 to 0.06)
LR_20–64_^a^	0.53 (− 0.4 to 1.46)	0.38 (− 0.33 to 1.08)	0.2 (− 0.22 to 0.61)	0.16 (− 0.1 to 0.42)	0.55 (− 0.22 to 1.32)	0.42 (− 0.18 to 1.01)	0.17 (−0.21 to 0.55)	0.17 (− 0.04 to 0.38)
P80/P20^a^	1.54^**^ (0.5 to 2.59)	1.2^**^ (0.4 to 1.99)	0.49^*^ (0.03 to 0.96)	0.32^*^ (0.03 to 0.61)	1.24^**^ (0.37 to 2.11)	0.85^*^ (0.17 to 1.52)	0.45^*^ (0.02 to 0.88)	0.18 (−0.05 to 0.42)
Taipei versus 4 counties^b^								
Sex (Male/Female)	2.38^***^ (1.93 to 2.83)	1.49^***^ (1.14 to 1.84)	0.71^***^ (0.53 to 0.9)	0.45^***^ (0.31 to 0.6)	2.07^***^ (1.69 to 2.44)	1.33^***^ (1.02 to 1.64)	0.64^***^ (0.48 to 0.81)	0.41^***^ (0.28 to 0.54)
Ln (GDP^a^ per capita), USD	−7.23^***^ (− 11.2 to − 3.26)	− 4.61^**^ (− 7.69 to − 1.52)	− 2.07^*^ (− 3.7 to −0.45)	− 2.47^***^ (− 3.7 to − 1.23)	−6.65^***^ (− 9.98 to − 3.32)	− 4.52^**^ (− 7.26 to − 1.78)	−1.91^*^ (− 3.37 to − 0.45)	− 2.26^***^ (− 3.39 to − 1.12)
Urbanization level (Taipei City/4 counties^b^)	−1.12^***^ (− 1.57 to − 0.67)	−1.16^***^ (− 1.5 to − 0.81)	−0.49^***^ (− 0.68 to − 0.31)	−0.35^***^ (− 0.5 to − 0.21)	−1.01^***^ (− 1.38 to − 0.63)	−1.02^***^ (− 1.33 to − 0.71)	−0.51^***^ (− 0.68 to − 0.35)	−0.26^***^ (− 0.39 to − 0.13)
Year 2008 (Yes/No)	−0.29 (− 1.29 to 0.72)	−0.37 (− 1.15 to 0.41)	−0.01 (− 0.42 to 0.4)	−0.29 (− 0.61 to 0.03)	−0.25 (− 1.09 to 0.59)	−0.2 (− 0.89 to 0.49)	−0.01 (− 0.38 to 0.36)	−0.21 (− 0.5 to 0.08)
Year 2009 (Yes/No)	−2.12^*^ (− 3.85 to − 0.39)	−1.25 (− 2.59 to 0.09)	−0.65 (− 1.36 to 0.06)	−0.6^*^ (− 1.16 to − 0.04)	−1.83^*^ (− 3.28 to − 0.38)	− 0.99 (− 2.18 to 0.2)	−0.51 (− 1.15 to 0.12)	−0.5^*^ (− 1 to − 0.01)
LR_20–64_^a^	1.32 (− 0.33 to 2.96)	0.85 (− 0.42 to 2.13)	0.47 (− 0.2 to 1.15	0.62^*^ (0.09 to 1.15)	1.51^*^ (0.13 to 2.89)	1.14^*^ (0 to 2.27)	0.49 (− 0.11 to 1.09)	0.69^**^ (0.22 to 1.16)
P80/P20^a^	1.98^*^ (0.13 to 3.83)	1.41 (− 0.03 to 2.85)	0.74 (− 0.01 to 1.5)	0.48 (− 0.11 to 1.07)	1.58^*^ (0.03 to 3.13)	0.93 (− 0.35 to 2.2)	0.63 (− 0.05 to 1.31)	0.32 (− 0.21 to 0.85)

^a^*CIR*_*16–99*_ cumulative incidence rate per 10^3^ person-years, aged 16–99, *GDP* gross domestic product, *MV* motor vehicle, *LR*_*20–64*_ literacy rate at age of 20–64 years, *P80/P20* income inequality based on the ratio of the average income of the richest 20% to the poorest 20%

^b^The 4 counties with the lowest population density: Taitung County, Yilan County, Hualien County, and Nantou County

Figures in parentheses are 95% confidence interval

Significant at *, **, and *** indicate significance at *p* < 0.05, 0.01, and 0.001, respectively

Previous studies revealed that poor residential or construction infrastructure was correlated with falls and fall-related injuries [45]. In Taiwan, the fact that more than 30% of patients with SCI were over 60 years of age (Table 1), of which the incidence rate would be higher because of a smaller denominator, deserves our attention. In fact, Taiwan government began to establish the first age-friendly city in Chia-Yi City in 2010 and has continued such efforts to build barrier-free environments in Taiwan at one of the fastest rates in the world [46]. The program might also contribute to the inverse association between incidence rate and fall-related SCI and GDP per capita in Taiwan (Table 3).

The global financial crisis between 2008 and 2009 was inversely associated with the occurrence of SCI, especially in 2009, because tourism activities probably declined over the recession period. In Greece, the mortality rate of MV accidents also fell by 45% in 2009 [47]. In this study, we also found that income inequality was positively associated with the occurrence of SCI, which corroborated with the results of previous studies [48]. The association was stronger in MV-related SCI than fall-related SCI, probably because a more equal distribution of income would improve citizen’s capability to purchase safety equipment for transportation (Fig. 1). Thus, reduction of income inequality through fiscal tools should also be included as a strategy to prevent SCI.

This study has the following limitations that must be acknowledged: First, since our NHI (National Health Insurance) claim data do not contain clinical details of AIS (ASIA (American Spinal Injury Association) Impairment Scale), we are unable to differentiate whether a patient is a complete or incomplete SCI. However, our registry of catastrophic illnesses stipulates that these patients must present with moderate to severe permanent functional disability to be eligible for this registry. Namely, we expected that all patients with complete SCI from severing trauma would be included. But our estimations cannot be generalized to SCI patients with mild or temporary functional impairment. Second, the promotion of age-friendly cities in Taiwan is generally based on the principle of aging in place and trying to adapt to heterogeneous local cultures. From 2013, the Ministry of Interior began to evaluate the quality of pedestrian barrier-free environment in every city every 2 years [49]. By assuming the trend of this score could be linearly extrapolated to 2002–2012, we added the new indicator into the original mixed-effects model. Although the score of pedestrian environment showed a consistent inverse effect on injury occurrences of tetraplegia, fall-related tetraplegia, and fall related SCI, it was statistically significant on CIR_16–99_ of tetraplegia alone (Supplementary Table 3), and we were unable to draw any strong inference. Future studies are needed to corroborate this hypothesis. Third, although this study brings attention to the importance of GDP per capita and infrastructures of transportation and construction, we have not explored the pathophysiology of individual SCI cases in details. More studies are warranted to develop effective prevention strategies for SCI occurrence on macro- and micro-levels to elevate economic performance, decrease income inequality, and improve infrastructures of transportation and construction, including age-friendly cities, barrier-free environment, and health literacy to adhere to safety regulations (Fig. 1).

## Conclusions

In conclusion, this study offers a comprehensive exploration of the trends and incidence rates of patients with tetraplegia of traumatic SCI in Taiwan. The associations between increased GDP per capita & reduced income inequality and reduced SCI incidence in Taiwan seem to have resulted from the provision of public goods and services, especially improvements in the infrastructure of transportation and construction, plus improved health literacy to adhere to regulations and individual financial ability to obtain safety equipment. In the coming years, we recommend that the government continues to improve infrastructure and enforce traffic regulations in rural regions of Taiwan to reduce the urban-rural disparity and decrease income inequality in the prevention of tetraplegia of traumatic SCI.

## Supplementary Information

**Additional file 1: Supplementary Table 1.** Estimation results of constructed mixed-effects model with CIR_16–59_† of spinal cord injury (SCI) as the dependent variable and major risk factors as fixed effects.

**Additional file 2: Supplementary Table 2.** Income effects of total spinal cord injury (SCI), traumatic SCI, motor vehicle (MV)-related SCI, fall-related SCI, tetraplegia, traumatic tetraplegia, MV-related tetraplegia, and fall-related tetraplegia in Taiwan.

**Additional file 3: Supplementary Table 3** Estimation results of mixed-effects model with CIR_16–99_† of spinal cord injury (SCI) as the dependent variable and major risk factors as fixed effects. The major risk factors in this mixed-effects model include one more indicator, the evaluation scores of pedestrian environment (SPE), to represent the quality of barrier-free environment.

## Data Availability

The Institutional Review Board (IRB) of National Cheng Kung University Hospital (NCKUH) are entitled and have full rights to oversee all activities, including the data of this study of each researcher, to comply with the Personal Data Protection Act. Anyone who is interested in analyzing the same dataset must write a formal proposal approved by the IRB of a university, institution, medical center, or a formal organization. Accompanied with the approval letter of the IRB of the researcher’s institution and the research proposal, the researcher can email the application to the Collaboration Center of Health Information Application, Ministry of Health and Welfare for access to the data.

## Abbreviations

SCI: Spinal cord injury
IR: Incidence rate
MV: Motor vehicle
US: United States
OECD: Organization for Economic Co-operation and Development
UK: United Kingdom
GDP: Gross domestic product
Ln (GDP per capita): Gross domestic product per capita in natural logarithm form
NHI: National Health Insurance
AMI: Acute myocardial infarction
COPD: Chronic obstructive pulmonary disease
ESRD: End-stage renal disease
CCI: Charlson Comorbidity Index
LR_20–64_: The literacy rate at 20–64 years
P80/P20: The ratio of the average income of the 20% richest to the 20% poorest
CIR_16–99_: Cumulative incidence rate per 10^3^ person-years, aged 16–99
CIR_16–59_: Cumulative incidence rate per 10^3^ person-years, aged 16–59
CI: Confidence interval
ICD-9-CM: International Classification of Disease, Ninth Revision, Clinical Modification

## References

[1] Sekhon LH, Fehlings MG (2001). Epidemiology, demographics, and pathophysiology of acute spinal cord injury. Spine..

[2] Huang H, Sun T, Chen L, Moviglia G, Chernykh E, von Wild K, Deda H, Kang KS, Kumar A, Jeon SR (2014). Consensus of clinical neurorestorative progress in patients with complete chronic spinal cord injury. Cell TransplantCell Transplant.

[3] Montoto-Marques A, Ferreiro-Velasco ME, Salvador-de la Barrera S, Balboa-Barreiro V, Rodriguez-Sotillo A, Meijide-Failde R (2017). Epidemiology of traumatic spinal cord injury in Galicia, Spain: trends over a 20-year period. Spinal CordSpinal Cord.

[4] Jain NB, Ayers GD, Peterson EN, Harris MB, Morse L, O'Connor KC, Garshick E (2015). Traumatic spinal cord injury in the United States, 1993-2012. JAMA..

[5] Chen HY, Chiu WT, Chen SS, Lee LS, Hung CI, Hung CL, Wang YC, Hung CC, Lin LS, Shih YH (1997). A nationwide epidemiological study of spinal cord injuries in Taiwan from July 1992 to June 1996. Neurol ResNeurol Res.

[6] Shingu H, Ohama M, Ikata T, Katoh S, Akatsu T (1995). A nationwide epidemiological survey of spinal cord injuries in Japan from January 1990 to December 1992. Spinal CordSpinal Cord.

[7] Aung TS, Masry WSE (1997). Audit of a British Centre for spinal injury. Spinal CordSpinal Cord.

[8] Martins F, Freitas F, Martins L, Dartigues JF, Barat M (1998). Spinal cord injuries – epidemiology in Portugal's central region. Spinal CordSpinal Cord.

[9] O'Connor P (2002). Incidence and patterns of spinal cord injury in Australia. Accid Anal Prev.

[10] Pagliacci MC, Celani MG, Zampolini M, Spizzichino L, Franceschini M, Baratta S, Finali G, Gatta G, Perdon L (2003). An Italian survey of traumatic spinal cord injury. Arch Phys Med RehabilArch Phys Med Rehabil.

[11] Selvarajah S, Hammond E, Haider A, Abularrage C, Becker D, Dhiman N, Hyder O, Gupta D, Black J, Schneider E (2014). The burden of acute traumatic spinal cord injury among adults in the United States: an update. J NeurotraumaJ Neurotrauma.

[12] Soopramanien A (1994). Epidemiology of spinal injuries in Romania. Spinal CordSpinal Cord.

[13] Silberstein B, Rabinovich S (1995). Epidemiology of spinal cord injuries in Novosibirsk, Russia. Spinal Cord.

[14] Maharaj JC (1996). Epidemiology of spinal cord paralysis in Fiji: 1985-1994. Spinal CordSpinal Cord.

[15] Singh A, Tetreault L, Kalsi-Ryan S, Nouri A, Fehlings MG (2014). Global prevalence and incidence of traumatic spinal cord injury. Clin Epidemiol.

[16] Jackson AB, Dijkers M, DeVivo MJ, Poczatek RB (2004). A demographic profile of new traumatic spinal cord injuries: change and stability over 30 years. Arch Phys Med RehabilArch Phys Med Rehabil.

[17] Oliver M, Inaba K, Tang A, Branco BC, Barmparas G, Schnuriger B, Lustenberger T, Demetriades D (2012). The changing epidemiology of spinal trauma: a 13-year review from a level I trauma Centre. Injury..

[18] Geisler WO, Jousse AT, Wynne-Jones M, Breithaupt D (1983). Survival in traumatic spinal cord injury. Spinal CordSpinal Cord.

[19] Manns PJ, Chad KE (2001). Components of quality of life for persons with a quadriplegic and paraplegic spinal cord injury. Qual Health ResQual Health Res.

[20] Ackers ML, Quick RE, Drasbek CJ, Hutwagner L, Tauxe RV (1998). Are there national risk factors for epidemic cholera? The correlation between socioeconomic and demographic indices and cholera incidence in Latin America. Int J EpidemiolInt J Epidemiol.

[21] van Beeck EF, Borsboom GJ, Mackenbach JP (2000). Economic development and traffic accident mortality in the industrialized world, 1962–1990. Int J EpidemiolInt J Epidemiol.

[22] Kumar R, Lim J, Mekary RA, Rattani A, Dewan MC, Sharif SY, Osorio-Fonseca E, Park KB (2018). Traumatic spinal injury: global epidemiology and worldwide volume. World Neurosurg.

[23] Braver ER (2003). Race, Hispanic origin, and socioeconomic status in relation to motor vehicle occupant death rates and risk factors among adults. Accid Anal Prev.

[24] Anbarci N, Escaleras M, Register CA (2009). Traffic fatalities: does income inequality create an externality?. Can J EconCan J Econ.

[25] McCaughey EJ, Purcell M, McLean AN, Fraser MH, Bewick A, Borotkanics RJ, Allan DB (2016). Changing demographics of spinal cord injury over a 20-year period: a longitudinal population-based study in Scotland. Spinal CordSpinal Cord.

[26] Lan C, Lai JS, Chang KH, Jean YC, Lien IN (1993). Traumatic spinal cord injuries in the rural region of Taiwan: an epidemiological study in Hualien county, 1986-1990. Spinal CordSpinal Cord.

[27] Jones T, Ugalde V, Franks P, Zhou H, White RH (2005). Venous thromboembolism after spinal cord injury: incidence, time course, and associated risk factors in 16,240 adults and children. Arch Phys Med RehabilArch Phys Med Rehabil.

[28] Thurman DJ, Sniezek JE, Johnson D, Greenspan A, Smith SM (1995). Guidelines for surveillance of central nervous system injury.

[29] National Health Insurance Administration, Ministry of Health and Welfare, Executive Yuan. 2014–2015 National Health Insurance Annual Report. 2016; Retrieved from https://www.nhi.gov.tw/Nhi_E-LibraryPubWeb/Periodical/Periodical.aspx?TML1_ID=4&Comm_Category_SN=0. [Accessed 01 Sep 2020].

[30] Lin YH, Tseng YH, Chen YC, Lin MH, Chou LF, Chen TJ, Hwang SJ (2013). The rural - urban divide in ambulatory care of gastrointestinal diseases in Taiwan. BMC Int Health Hum RightsBMC Int Health Hum Rights.

[31] Griffiths RI, O'Malley CD, Herbert RJ, Danese MD (2013). Misclassification of incident conditions using claims data: impact of varying the period used to exclude pre-existing disease. BMC Med Res MethodolBMC Med Res Methodol.

[32] Romano PS, Roos LL, Jollis JG (1993). Adapting a clinical comorbidity index for use with ICD-9-CM administrative data: differing perspectives. J Clin EpidemiolJ Clin Epidemiol.

[33] Toda M, Nakatani E, Omae K, Fukushima M, Chin T (2018). Age-specific characterization of spinal cord injuries over a 19-year period at a Japanese rehabilitation center. PLoS OnePLoS One.

[34] Day NE, Waterhouse JW, Muir CS, Correa P, Powell J (1976). A new measure of age standardized incidence, the cumulative rate. Cancer Incidence in Five Continents, Volume III. IARC Scientific Publications No. 15.

[35] Directorate-General of Budget, Accounting and Statistics, Executive Yuan. Gross domestic product and economic growth rate. 2019; Retrieved from https://www.dgbas.gov.tw/ct.asp?xItem=44245&ctNode=3339&mp=1. [Accessed 23 Nov 2019].

[36] Department of Statistics, Ministry of the Interior, Executive Yuan. Levels of educational attainment for age 15 plus. 2019; Retrieved from http://statis.moi.gov.tw/micst/stmain.jsp?sys=100&kind=10&type=1&funid=c01104&rdm=lheeJ7py. [Accessed 01 Sep 2020].

[37] Directorate-General of Budget, Accounting and Statistics, Executive Yuan. Report on the survey of family income and expenditure, 2018. 2019; Retrieved from https://win.dgbas.gov.tw/fies/a11.asp?year=107. [Accessed 01 Sep 2020].

[38] Newhouse JP (1977). Medical-care expenditure: a cross-national survey. J Hum ResourJ Hum Resour.

[39] Chen Y, Tang Y, Vogel LC, DeVivo MJ (2013). Causes of spinal cord injury. Top Spinal Cord Inj Rehabil.

[40] Chen CF, Lien IN (1985). Spinal cord injuries in Taipei, Taiwan, 1978-1981. Spinal CordSpinal Cord.

[41] Kim E (2002). Determinants of optimal level of transportation infrastructure. J Urban Plan Dev.

[42] Department of Statistics, Ministry of Transportation and Communications. The analysis of statistical abstract of daily transport. 2017; Retrieved from https://www.motc.gov.tw/ch/home.jsp?id=54&parentpath=0,6. [Accessed 23 Nov 2019].

[43] Lan LW, Wang MT, Kuo AY (2006). Development and deployment of public transport policy and planning in Taiwan. Transportation..

[44] Ministry of Transportation and Communications. Statistical Abstract of Transportation and Communications, Republic of China 2002–2015. 2015; Retrieved from https://www.motc.gov.tw/ch/home.jsp?id=2050&parentpath=0%2C6&mcustomize=statistics501.jsp. [Accessed 23 Nov 2019].

[45] Hoffman GJ, Rodriguez HP (2015). Examining contextual influences on fall-related injuries among older adults for population health management. Popul Health Manag.

[46] Lin YY, Huang CS (2015). Aging in Taiwan: building a society for active aging and aging in place. Gerontologist..

[47] Mpogas K, Kopelias P, Mitropoulos L, Kepaptsoglou K (2017). Road safety in urban areas in Greece during economy downturn. A before – after comparison. Transp Res Proc.

[48] Roshanfekr P, Khodaie-Ardakani M-R, Sajjadi H, Malek Afzali Ardakani H (2020). Income-related inequality in traffic accident health outcomes (injury, disability and mortality): evidence from the nationwide survey in Iran. Iran J Public HealthIran J Public Health.

[49] Construction and Planning Agency, Ministry of the Interior. Evaluation of pedestrian environment. 2015; Retrieved from https://myway.cpami.gov.tw/. [Accessed 01 Dec 2020].

