# Supplementary material for: The association between economic indicators and the incidence of tetraplegia from traumatic spinal cord injury in Taiwan
Source: BMC Neurol. 2021 Mar 17;21:117. doi: 10.1186/s12883-021-02141-8 (PMC7968275; doi:10.1186/s12883-021-02141-8)
Supplement: Supplementary file 1 — Additional file 1: Supplementary Table 1. Estimation results of constructed mixed-effects model with CIR16–59† of spinal cord injury (SCI) as the dependent variable and major risk factors as fixed effects. [file 12883_2021_2141_MOESM1_ESM.docx]

**Supplementary Table 1** Estimation results of constructed mixed-effects model with CIR_16−59_† of spinal cord injury (SCI) as the dependent variable and major risk factors as fixed effects.

|  | Total SCI | Traumatic SCI | MV-related SCI | Fall-related SCI | Tetraplegia | Traumatic tetraplegia | MV-related tetraplegia | Fall-related tetraplegia |
| --- | --- | --- | --- | --- | --- | --- | --- | --- |
| National data |  |  |  |  |  |  |  |  |
| Sex (Male/Female) | 0.86^***^ (0.76 to 0.97) | 0.57^***^ (0.5 to 0.64) | 0.28^***^ (0.24 to 0.32) | 0.18^***^ (0.15 to 0.21) | 0.6^***^ (0.52 to 0.68) | 0.4^***^ (0.34 to 0.45) | 0.22^***^ (0.18 to 0.26) | 0.12^***^ (0.1 to 0.13) |
| Ln(GDP† per capita), USD | −2^***^ (−2.9 to −1.11) | −1.26^***^ (−1.89 to −0.62) | −0.62^**^ (−1.01 to −0.23) | −0.4^**^ (−0.65 to −0.15) | −1.23^***^ (−1.91 to −0.54) | −0.78^**^ (−1.26 to −0.3) | −0.51^**^ (−0.85 to −0.16) | −0.16 (−0.33 to 0) |
| Urbanization level (Metropolitan/Rural) | −0.21^***^ (−0.31 to −0.1) | −0.17^***^ (−0.24 to −0.1) | −0.1^***^ (−0.15 to −0.06) | −0.05^**^ (−0.08 to −0.02) | −0.15^***^ (−0.22 to −0.07) | −0.13^***^ (−0.18 to −0.07) | −0.09^***^ (−0.13 to −0.05) | −0.03^**^ (−0.05 to −0.01) |
| Year 2008 (Yes/No) | −0.04 (−0.27 to 0.18) | −0.05 (−0.21 to 0.11) | −0.02 (−0.12 to 0.08) | −0.03 (−0.09 to 0.04) | 0.03 (−0.14 to 0.2) | 0 (−0.12 to 0.12) | 0 (−0.09 to 0.08) | 0 (−0.04 to 0.04) |
| Year 2009 (Yes/No) | −0.48^*^ (−0.87 to −0.09) | −0.4^**^ (−0.67 to −0.12) | −0.19^*^ (−0.36 to −0.02) | −0.13^*^ (−0.24 to −0.01) | −0.33^*^ (−0.63 to −0.03) | −0.25^*^ (−0.46 to −0.04) | −0.15 (−0.3 to 0) | −0.06 (−0.13 to 0.02) |
| LR_20-64_† | 0.16 (−0.21 to 0.52) | 0.11 (−0.15 to 0.37) | 0.03 (−0.13 to 0.19) | 0.06 (−0.05 to 0.16) | 0.11 (−0.17 to 0.4) | 0.09 (−0.11 to 0.29) | 0.06 (−0.08 to 0.21) | 0.02 (−0.05 to 0.08) |
| P80/P20† | 0.36 (−0.05 to 0.78) | 0.33^*^ (0.04 to 0.63) | 0.12 (−0.07 to 0.3) | 0.12 (0 to 0.24) | 0.3 (−0.02 to 0.61) | 0.22 (0 to 0.45) | 0.1 (−0.06 to 0.26) | 0.06 (−0.01 to 0.14) |
| Taipei versus 4 counties‡ |  |  |  |  |  |  |  |  |
| Sex (Male/Female) | 1.05^***^ (0.88 to 1.22) | 0.69^***^ (0.57 to 0.82) | 0.3^***^ (0.22 to 0.39) | 0.25^***^ (0.2 to 0.3) | 0.75^***^ (0.63 to 0.87) | 0.51^***^ (0.41 to 0.62) | 0.24^***^ (0.17 to 0.32) | 0.18^***^ (0.12 to 0.23) |
| Ln(GDP† per capita), USD | −2.12^**^ (−3.66 to −0.58) | −0.94 (−2.06 to 0.19) | −0.39 (−1.15 to 0.37) | −0.72^**^ (−1.15 to −0.29) | −1.57^**^ (−2.65 to −0.49) | −0.92 (−1.83 to 0) | −0.58 (−1.23 to 0.07) | −0.34 (−0.81 to 0.13) |
| Urbanization level (Taipei City/4 counties‡) | −0.32^***^ (−0.5 to −0.15) | −0.31^***^ (−0.44 to −0.18) | −0.15^***^ (−0.24 to −0.07) | −0.09^***^ (−0.14 to −0.04) | −0.22^***^ (−0.35 to −0.1) | −0.23^***^ (−0.33 to −0.13) | −0.15^***^ (−0.22 to −0.08) | −0.04 (−0.1 to 0.01) |
| Year 2008 (Yes/No) | 0.03 (−0.36 to 0.41) | −0.06 (−0.35 to 0.22) | 0.03 (−0.16 to 0.22) | −0.08 (−0.2 to 0.03) | 0.1 (−0.17 to 0.38) | 0.01 (−0.22 to 0.24) | 0 (−0.16 to 0.17) | −0.02 (−0.14 to 0.1) |
| Year 2009 (Yes/No) | −0.86^*^ (−1.52 to −0.19) | −0.71^**^ (−1.2 to −0.22) | −0.26 (−0.59 to 0.07) | −0.31^**^ (−0.51 to −0.12) | −0.57^*^ (−1.05 to −0.1) | −0.45^*^ (−0.84 to −0.05) | −0.24 (−0.52 to 0.04) | −0.15 (−0.36 to 0.05) |
| LR_20-64_† | 0.93 (−0.63 to 0.64) | −0.08 (−0.54 to 0.39) | −0.02 (−0.33 to 0.3) | 0.12 (−0.06 to 0.31) | 0.14 (−0.31 to 0.59) | 0.15 (−0.23 to 0.53) | 0.12 (−0.15 to 0.39) | 0.06 (−0.14 to 0.25) |
| P80/P20† | 0.64 (−0.07 to 1.36) | 0.7^*^ (0.17 to 1.22) | 0.26 (−0.09 to 0.62) | 0.28^**^ (0.07 to 0.49) | 0.35 (−0.15 to 0.86) | 0.34 (−0.09 to 0.76) | 0.19 (−0.12 to 0.49) | 0.13 (−0.09 to 0.35) |

†CIR_16−59_: cumulative incidence rate per 10^3^ person-years, aged 16−59; GDP: gross domestic product; SCI: spinal cord injury; MV: motor vehicle; LR_20-64_: literacy rate at age of 20-64 years; P80/P20: income inequality based on the ratio of the average income of the 20% richest to the 20% poorest.

‡The 4 counties with the lowest population density: Taitung County, Yilan County, Hualien County, and Nantou County.

Figures in parentheses are 95% confidence interval.

Significant at *, **, and *** indicate significance at *p*<0.05, 0.01, and 0.001, respectively.
