# Supplementary material for: The association between economic indicators and the incidence of tetraplegia from traumatic spinal cord injury in Taiwan
Source: BMC Neurol. 2021 Mar 17;21:117. doi: 10.1186/s12883-021-02141-8 (PMC7968275; doi:10.1186/s12883-021-02141-8)
Supplement: Supplementary file 2 — Additional file 2: Supplementary Table 2. Income effects of total spinal cord injury (SCI), traumatic SCI, motor vehicle (MV)-related SCI, fall-related SCI, tetraplegia, traumatic tetraplegia, MV-related tetraplegia, and fall-related tetraplegia in Taiwan. [file 12883_2021_2141_MOESM2_ESM.docx]

**Supplementary Table 2** Income effects of total spinal cord injury (SCI), traumatic SCI, motor vehicle (MV)-related SCI, fall-related SCI, tetraplegia, traumatic tetraplegia, MV-related tetraplegia, and fall-related tetraplegia in Taiwan.

|  | Total SCI | Traumatic SCI | MV-related SCI | Fall-related SCI | Tetraplegia | Traumatic tetraplegia | MV-related tetraplegia | Fall-related tetraplegia |
| --- | --- | --- | --- | --- | --- | --- | --- | --- |
| CIR_16−99_ (95% CI), ‰ | 3.5  (2.9 to 4.2) | 2.3  (1.9 to 2.7) | 1.0  (0.7 to 1.1) | 0.8  (0.6 to 1.0) | 2.5  (2.0 to 2.9) | 1.6  (1.3 to 1.9) | 0.8  (0.6 to 0.9) | 0.5  (0.4 to 0.7) |
| Income elasticity | −1.39 | −1.34 | −1.55 | −1.36 | −1.46 | −1.54 | −1.54 | −1.62 |

CIR_16−99_: cumulative incidence rate per 10^3^ person-years, aged 16−99 years; CI: confidence interval.
