# Supplementary material for: The association between economic indicators and the incidence of tetraplegia from traumatic spinal cord injury in Taiwan
Source: BMC Neurol. 2021 Mar 17;21:117. doi: 10.1186/s12883-021-02141-8 (PMC7968275; doi:10.1186/s12883-021-02141-8)
Supplement: Supplementary file 3 — Additional file 3: Supplementary Table 3 Estimation results of mixed-effects model with CIR16–99† of spinal cord injury (SCI) as the dependent variable and major risk factors as fixed effects. The major risk factors in this mixed-effects model include one more indicator, the evaluation scores of pedestrian environment (SPE), to represent the quality of barrier-free environment. [file 12883_2021_2141_MOESM3_ESM.docx]

**Supplementary Table 3** Estimation results of mixed-effects model with CIR_16−99_† of spinal cord injury (SCI) as the dependent variable and major risk factors as fixed effects. The major risk factors in this mixed-effects model include one more indicator, the evaluation scores of pedestrian environment (SPE), to represent the quality of barrier-free environment.

| Taipei versus 4 counties |  | Sex (Male/Female) | Ln(GDP† per capita), USD | Urbanization level (Taipei City/4 counties ‡) | Year 2008 (Yes/No) | Year 2009 (Yes/No) | LR_20-64_† | P80/P20† | SPE |
| --- | --- | --- | --- | --- | --- | --- | --- | --- | --- |
|  | Fall-related SCI | 0.45*** (0.31 to 0.59) | –1.87* (–3.38 to –0.36) | 0.01 (–0.54 to 0.56) | –0.27 (–0.59 to 0.04) | –0.49 (–1.07 to 0.08) | 0.58* (0.05 to 1.11) | 0.42 (–0.18 to 1.02) | –0.02 (–0.05 to 0.01) |
|  | Tetraplegia | 2.07*** (1.71 to 2.42) | –3.95* (–7.77 to –0.13) | 0.65 (–0.72 to 2.02) | –0.19 (–0.99 to 0.61) | –1.35 (–2.78 to 0.07) | 1.36* (0.05 to 2.67) | 1.3 (–0.18 to 2.79) | –0.1* (–0.18 to –0.02) |
|  | Fall-related tetraplegia | 0.41*** (0.29 to 0.54) | –1.72* (–3.09 to –0.36) | 0.06 (–0.42 to 0.55) | –0.2 (–0.48 to 0.09) | –0.41 (–0.92 to 0.1) | 0.66** (0.19 to 1.13) | 0.26 (–0.27 to 0.79) | –0.02 (–0.05 to 0.01) |

†CIR_16−99_: cumulative incidence rate per 10^3^ person-years, aged 16−99; GDP: gross domestic product; MV: motor vehicle; LR_20-64_: literacy rate at age of 20-64 years; P80/P20: income inequality based on the ratio of the average income of the richest 20% to the poorest 20%.

‡The 4 counties with the lowest population density: Taitung County, Yilan County, Hualien County, and Nantou County.

Figures in parentheses are 95% confidence interval.

Significant at *, **, and *** indicate significance at *p*<0.05, 0.01, and 0.001, respectively.
